# Supplementary material for: Infection-sensing minigenome as a novel therapeutic approach against Ebola virus
Source: Mol Ther Nucleic Acids. 2025 Sep 22;36(4):102722. doi: 10.1016/j.omtn.2025.102722 (PMC12546956; doi:10.1016/j.omtn.2025.102722)
Supplement: Document S1. Figure S1 [file mmc1.pdf]

OMTN, Volume *36*

Supplemental information

Infection-sensing minigenome as a novel therapeutic approach against Ebola virus

Lin Wang, Brady N. Zell, Brian J. Parrett, Michael A. Barry, and Satoko Yamaoka

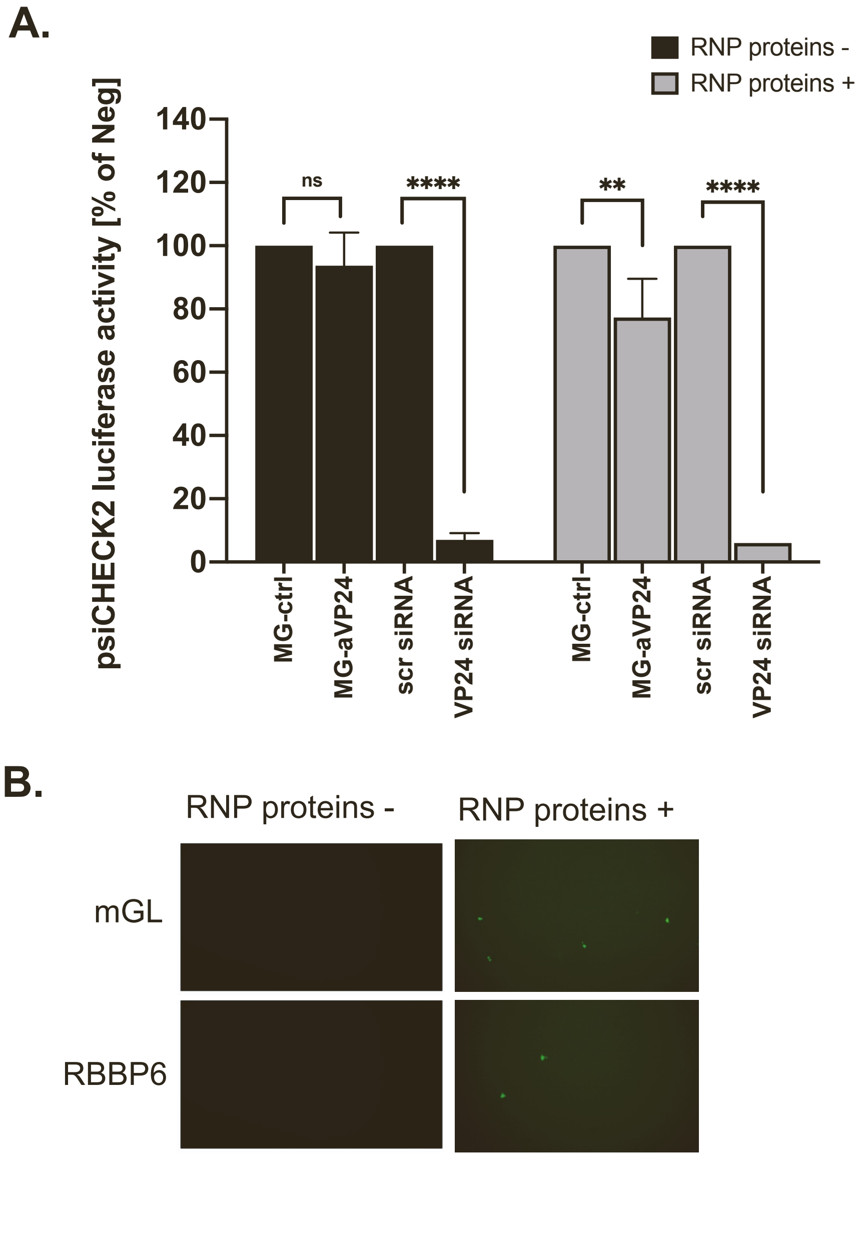
A.
RNP proteins -
RNP proteins +
psiCHECK2 luciferase activity [% of Neg]
140
120
100
80
60
40
20
0
ns
****
**
****
MG-ctrl
MG-aVP24
scr siRNA
VP24 siRNA
MG-ctrl
MG-aVP24
scr siRNA
VP24 siRNA
B.
RNP proteins -
RNP proteins +
mGL
RBBP6

Fig. S1. Stability of therapeutic RNAs. A) The IVT 1cis-MG-ctrl and 1cis-MG-aVP24 RNAs, scramble and anti-VP24 siRNAs, were transfected into 293 cells 24 hours before the transfection of EBOV helper plasmids (pCAGGS-L, VP30, VP35 and NP). Six hours after helper plasmids transfection, the psiCHECK2-VP24 plasmid was introduced to evaluate the silencing of VP24. Luciferase activity was measured 24 hours post-transfection. *Renilla* luciferase activity was normalized to firefly luciferase activity. Data are shown as mean percentage reduction of luciferase activity (*Renilla*/firefly) relative to each negative control, set at 100%. Error bars indicate the SD of three independent biological replicates. Ordinary One-way ANOVA was used for statistical analysis: ns: $P > 0.05$, ** $P \leq 0.01$, **** $P \leq 0.0001$. **B**) IVT 1cis-MG-mGL and 1cis-MG-mGL-RBBP6 RNAs were transfected into 293 cells 24 hours prior to EBOV helper plasmids transfection. Fluorescence images (original magnification x250) were taken 24 hours after helper plasmids transfection.
