# Supplementary material for: Infection-sensing minigenome as a novel therapeutic approach against Ebola virus
Source: Mol Ther Nucleic Acids. 2025 Sep 22;36(4):102722. doi: 10.1016/j.omtn.2025.102722 (PMC12546956; doi:10.1016/j.omtn.2025.102722)
Supplement: Document S2. Article plus supplemental information [file mmc2.pdf]

Molecular Therapy
Nucleic Acids
Original Article

Infection-sensing minigenome as a novel therapeutic approach against Ebola virus

Lin Wang,[1] Brady N. Zell,[2] Brian J. Parrett,[2] Michael A. Barry,[1,3,4] and Satoko Yamaoka[1,5]

[1]Division of Infectious Diseases, Department of Medicine, Mayo Clinic, Rochester, MN, USA; [2]Virology and Gene Therapy Program, Mayo Clinic Graduate School of Biomedical Sciences, Rochester, MN, USA; [3]Department of Immunology, Mayo Clinic, Rochester, MN, USA; [4]Department of Molecular Medicine, Mayo Clinic, Rochester, MN, USA

We describe here a molecular therapy that uses the virus's own proteins to combat itself. In this approach, infection-sensing RNAs encoding therapeutic genes are flanked by viral promoters and packaging signals in negative-sense orientation. These therapeutic minigenome RNAs do not express the transgene and remain silent in the absence of a viral infection. In contrast, if the cell is infected, the virus acts as a helper virus, providing viral proteins *in trans* to transcribe, replicate, and package the therapeutic minigenomes. Proof of concept for this therapeutic approach is demonstrated here using Ebola virus (EBOV) minigenomes expressing two antiviral transgenes: a short-hairpin (sh) RNA targeting the EBOV viral protein VP24 and an open-reading frame expressing a host peptide from the retinoblastoma-binding protein 6 (RBBP6). By using an EBOV tetracistronic-minigenome as a virus life cycle modeling system, we show here that both therapeutic minigenomes suppressed viral RNA replication and viral protein production, and a more than 50% reduction in the reporter signal was observed when cells were challenged with EBOV transcription/replication-competent virus-like particles. These findings highlight the therapeutic potential of infection-sensing minigenome to combat EBOV and perhaps other viral pathogens.

INTRODUCTION

Ebola virus (EBOV) is an enveloped, non-segmented, negative-sense RNA virus in the family *Filoviridae.* Among the deadliest of emerging or already emerged viruses, EBOV causes severe febrile illness and frequently leads to death within ten days of infection due to multi-organ failure.[1–8] The severity of this infectious agent became most evident during the 2014–2016 outbreak in Western Africa, which lead to over 28,000 confirmed cases and more than 11,000 fatalities.[9] Given the dire threat of EBOV and other filoviruses to humans, a broad array of therapies have been developed including small-molecule drugs, monoclonal antibodies (mAbs), and viral vaccine vectors.[10] While these diverse antiviral agents each hold great promise, they also have limitations.

Small-molecule antiviral drugs have proven to be effective in combating viral infections,[11,12] with some even having anti-filoviral potential.[12,13] Small molecules frequently carry the risk of inducing off-target effects and antiviral resistance due to selective pressure.[14,15] mAbs against EBOV glycoprotein (GP) have also demonstrated potential. Two mAb therapies, Inmazeb (REGN-EB3) and Ebanga (mAb114), are approved for the treatment of EBOV disease (EVD).[10,16–18] mAbs have an excellent target selectivity and reduced toxicity compared to small molecules. However, they also come with certain limitations, including production costs, issues with tissue penetration, and short serum half-life.[19,20] Additionally, it has been noted that mAb treatment could hinder the production of host-produced, anti-EBOV antibodies in individuals who have survived EVD, which might risk reinfection or reactivation of the virus.[21] An EBOV vaccine, ERVEBO, is a replication-competent recombinant vesicular-stomatitis virus (VSV) pseudotyped with the EBOV GP which has also shown efficacy in post-exposure prophylaxis.[22,23] However, the disadvantages of this vaccine include viral shedding of VSV and potential adverse effects including fatigue, headache, fever, and muscle pain.[24,25] Another EBOV vaccine which uses a replication-defective chimpanzee adenovirus 3 (ChAd3) vector to express the Zaire EBOVGP (ChAd3-EBO-Z), and is boosted with modified vaccinia Ankara, is primarily designed to protect against EBOV.[26]

Our group generated a novel antiviral molecular therapy, referred to as the "therapeutic minigenome (MG)." This approach utilizes a therapeutic transgene flanked by the 3′ and 5′ ends of the natural viral genome which contains crucial signals for genome replication, transcription, and encapsidation. Once the viral polymerase recognizes these signals, the therapeutic MG is replicated and transcribed

Received 6 February 2025; accepted 19 September 2025;
https://doi.org/10.1016/j.omtn.2025.102722.

[5]Present address: HDT Bio, Seattle, WA, USA

Correspondence: Lin Wang, Division of Infectious Diseases, Department of Medicine, Mayo Clinic, Rochester, MN, USA.
E-mail: wang.lin@mayo.edu
Correspondence: Michael A. Barry, Division of Infectious Diseases, Department of Medicine, Mayo Clinic, Rochester, MN, USA.
E-mail: barry.michael@mayo.edu
Correspondence: Satoko Yamaoka, Division of Infectious Diseases, Department of Medicine, Mayo Clinic, Rochester, MN, USA.
E-mail: satoko.yamaoka@hdt.bio

© 2025 The Author(s). Published by Elsevier Inc. on behalf of The American Society of Gene and Cell Therapy.
This is an open access article under the CC BY license (http://creativecommons.org/licenses/by/4.0/).

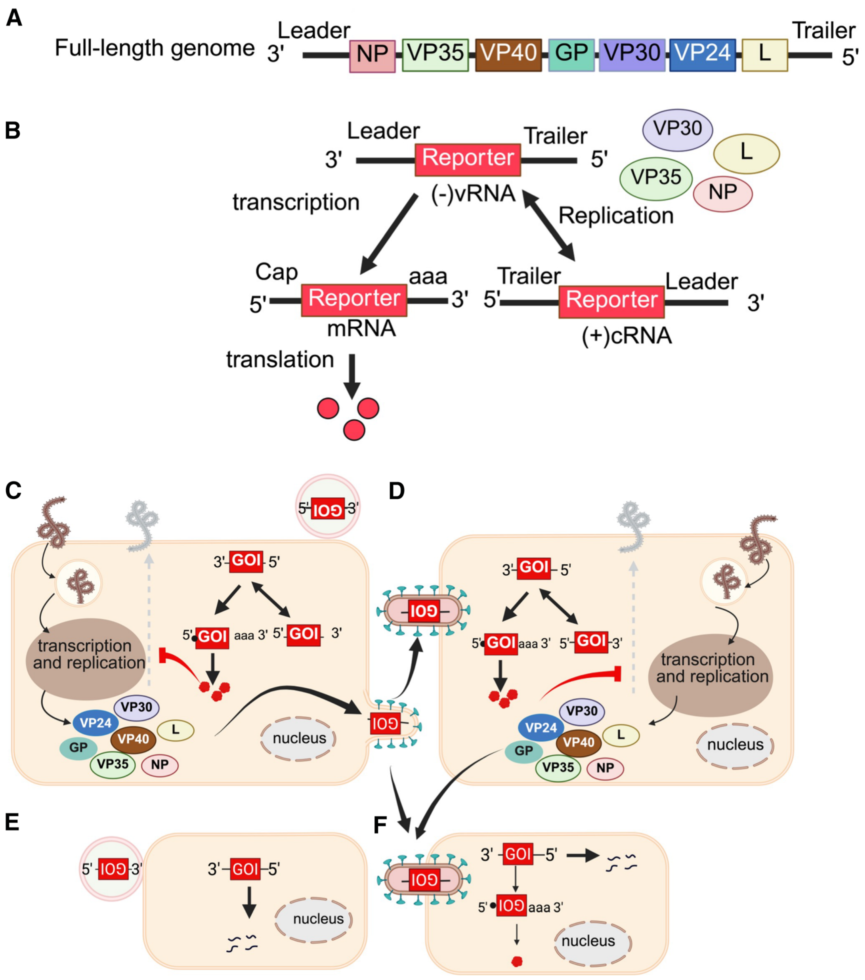

Figure 1. Scheme of minigenome therapeutic approach

(A) Full-length EBOV genome. (B) Replication and translation of minigenome therapeutic transgene cassette. (C) Delivery of MG RNA by transfection to an EBOV-infected cell. (D) Delivery of MG RNA by trVLPs to an EBOV-infected target cell. (E) Delivery of MG RNA by transfection to a non-infected cell. (F) Delivery of MG RNA by trVLPs to a non-infected target cell. GOI, gene of interest (therapeutic transgene).

by viral ribonucleoprotein (RNP) complex proteins.[27–29] Since the 3′ and 5′ ends also contain genome packaging signals, these amplified MGs can be packaged into transcription/replication-competent virus-like particles (trVLPs).[30] After budding from the cell, these outgoing trVLPs can infect a second wave of target cells, thereby establishing a unique mechanism for the targeted self-delivery of therapeutics. Importantly, the proposed therapeutic MG is designed on a negative-sense RNA platform, ensuring that therapeutic genes are not expressed in healthy cells, and are only amplified and expressed in virus-infected cells.

As a proof-of-concept, we utilize the EBOV MG system as a model to express two types of antiviral transgenes: shRNA targeting the EBOV viral protein VP24 and a peptide from the human antiviral protein, retinoblastoma binding protein 6 (RBBP6).

RESULTS

Scheme of therapeutic MG targeting EBOV

EBOV has a single-stranded, negative-sense RNA genome which contains seven genes encoding for structural proteins, including nucleoprotein (NP), polymerase cofactor VP35, matrix protein VP40, surface glycoprotein (GP), transcriptional activator VP30, genome packaging factor VP24, and the RNA-dependent RNA-polymerase (L) (Figure 1A).[31] The termini of the genome are untranslated regions (UTRs) containing 3′ leader and 5′ trailer sequences which direct replication, transcription, and encapsidation.[29,30,32]

The proposed therapeutic MG is designed to encode an antiviral transgene flanked by the EBOV 3′ leader and 5′ trailer in the negative-sense orientation (Figure 1B). In the presence of EBOV infection, the therapeutic MG RNA is replicated and transcribed through the functions of EBOV-RNP complex proteins, including NP, VP35, VP30, and L, ultimately leading to therapeutic gene expression. Viral ribonucleocapsids containing newly synthesized MG RNA are then condensed by VP24 and packaged into trVLPs formed by VP40 (Figure 1C). The released trVLPs display EBOV GP on their surface allowing them to deliver the therapeutic MG RNA to EBOV target cells (Figure 1D). These amplification cycles continue as long as the proteins supplied by EBOV are present. In non-infected cells, the therapeutic MG RNA remains silent and is eventually degraded, resulting in no therapeutic gene expression (Figure 1E). When the MG RNA is delivered to cells via trVLPs, it can be transcribed by the RNP proteins that were packaged with the MG RNA. However, replication will not occur unless the cell later becomes infected with EBOV (Figure 1F). In this study, the therapeutic MG was designed to encode a single transgene, referred to as a monocistronic-MG (1*cis*-MG).

Therapeutic MG-encoding anti-VP24 shRNA is transcribed and facilitates VP24 silencing with EBOV RNP proteins supplied *in trans*

Previous studies have shown that the EBOV VP24 protein is essential for the incorporation of full-length EBOV genomes and polycistronic MGs into viral particles, but is dispensable for the incorporation of short viral genomes like the 1*cis*-MG.[33,34] We therefore

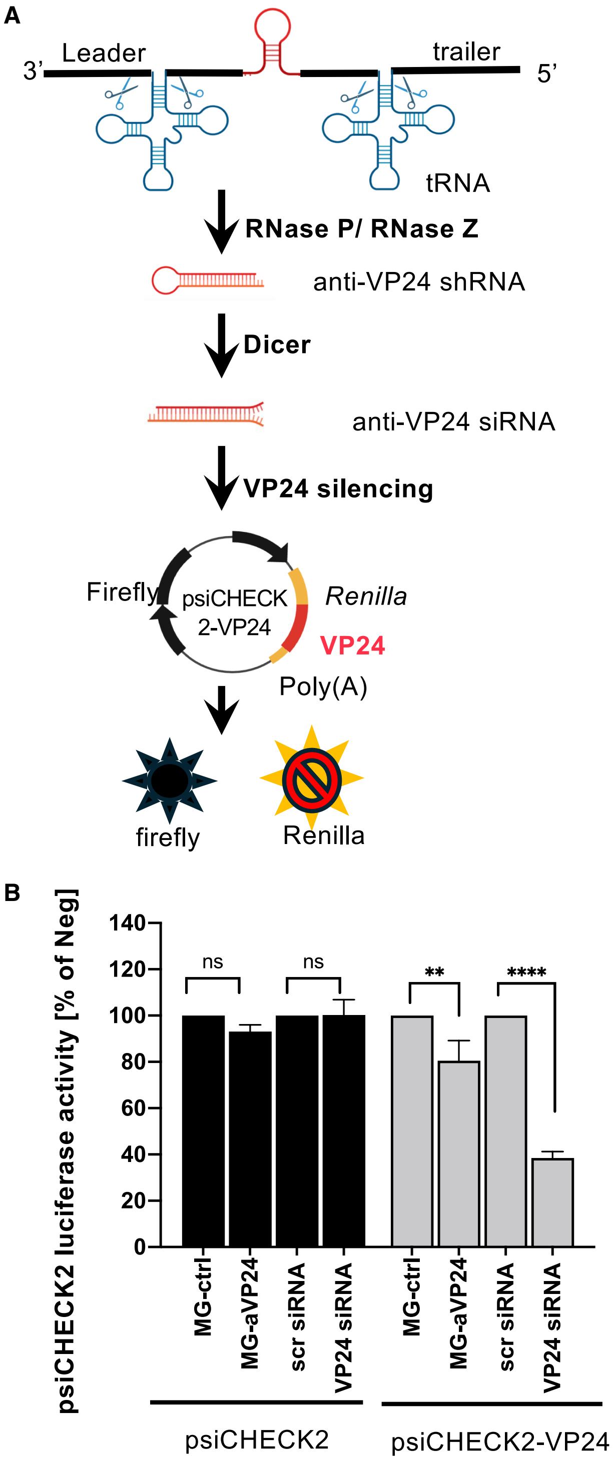

Figure 2. Expression of 1*cis*-MG-anti-VP24 and silencing of VP24

(A) Schematic illustrations of the working mechanisms of 1*cis*-MG-anti-VP24. (B) The indicated plasmids and RNAs were transfected into 293 cells and luciferase activity was measured 24 h later. The cells were also co-transfected with plasmids expressing EBOV helper proteins. *Renilla* luciferase activity was normalized with firefly luciferase activity. Data are shown as mean percentage reduction of luciferase activity (*Renilla*/firefly) relative to each negative control, which was set at 100%. MG-ctrl: 1*cis*-MG vector. scr siRNA: scramble siRNA. Error bars represent SD of three independent biological replicates. Ordinary One-way ANOVA was used for statistical analysis: ns, $p > 0.05$, **$p \leq 0.01$, ****$p \leq 0.0001$. If there was no statistical significance for a given comparison, it was indicated as "ns."

hypothesized that targeting VP24 could selectively inhibit the production of infectious EBOV, while having minimal effect on the packaging of therapeutic 1*cis*-MG in trVLPs. To achieve this objective, we first generated a T7 polymerase-driven 1*cis*-MG expression plasmid that encodes an anti-VP24 shRNA sequence as a therapeutic payload.[35,36] Specifically, a single copy of anti-VP24 siRNA sequence, flanked by precursor tRNA (pre-tRNA) sequences, was introduced between the conserved EBOV 3′ and 5′ ends (Figure 2A).[37–39] This design enables the cleavage of VP24 shRNA from viral transcripts via pre-tRNA cleavage mediated by RNases P and Z.[40,41] The resulting anti-VP24 shRNA can be further processed by Dicer to generate anti-VP24 siRNA (Figure 2A).[42]

The silencing effect of VP24 siRNA expressed from 1*cis*-MG-anti-VP24 was evaluated using a dual-luciferase assay with psiCHECK2-VP24 containing the VP24 target sequence fused to *Renilla* luciferase (Figure 2A). The functionality of this system was validated by a significant reduction (approximately 60%) in luciferase activity upon expressing 25 nM of synthetic anti-VP24 siRNA compared with the scrambled siRNA control (Figure 2B). When the 1*cis*-MG-anti-VP24 plasmid was co-transfected with psiCHECK2-VP24, this resulted in 20% reduction in luciferase activity in the presence of EBOV RNP proteins when compared to co-transfection with the empty 1*cis*-MG plasmid. The inhibitory effect was observed for MG-anti-VP24 IVT RNA up to 24h before transfection of helper plasmids (Figure S1A). No significant reduction in luciferase activity was observed when the psiCHECK2 plasmid lacking the VP24 sequence was supplied in the system. These results provide the first evidence that the negative-sense MG RNA can be used to express siRNAs, effectively suppressing target gene expression in a sequence-specific manner.

In vitro transcribed therapeutic MG RNA-encoding anti-VP24 shRNA amplifies in the presence of EBOV RNP proteins

Using the 1*cis*-MG-anti-VP24 plasmid as a template, 1*cis*-MG-anti-VP24 RNA was generated by *in vitro* transcription (IVT) by T7 RNA polymerase (Figure 3A). The synthesized MG RNA was resolved on a 0.8% agarose gel by electrophoresis, where it appeared as a distinct band corresponding to the expected size (Figure 3B). To test whether 1*cis*-MG-anti-VP24 RNA can replicate in cells, 293 cells were co-transfected with IVT MG RNA and helper plasmids expressing RNP proteins. The quantity of MG RNA was measured by RT-qPCR using primers specific to pre-tRNA and leader sequences on the 1*cis*-MG-anti-VP24 RNA (Figure 3C). The RT-qPCR result showed a significant increase in MG RNA copies from 6 to 24h after transfection (Figure 3D), suggesting that 1*cis*-MG-anti-VP24 IVT RNA was replicated in the presence of RNP complex proteins.

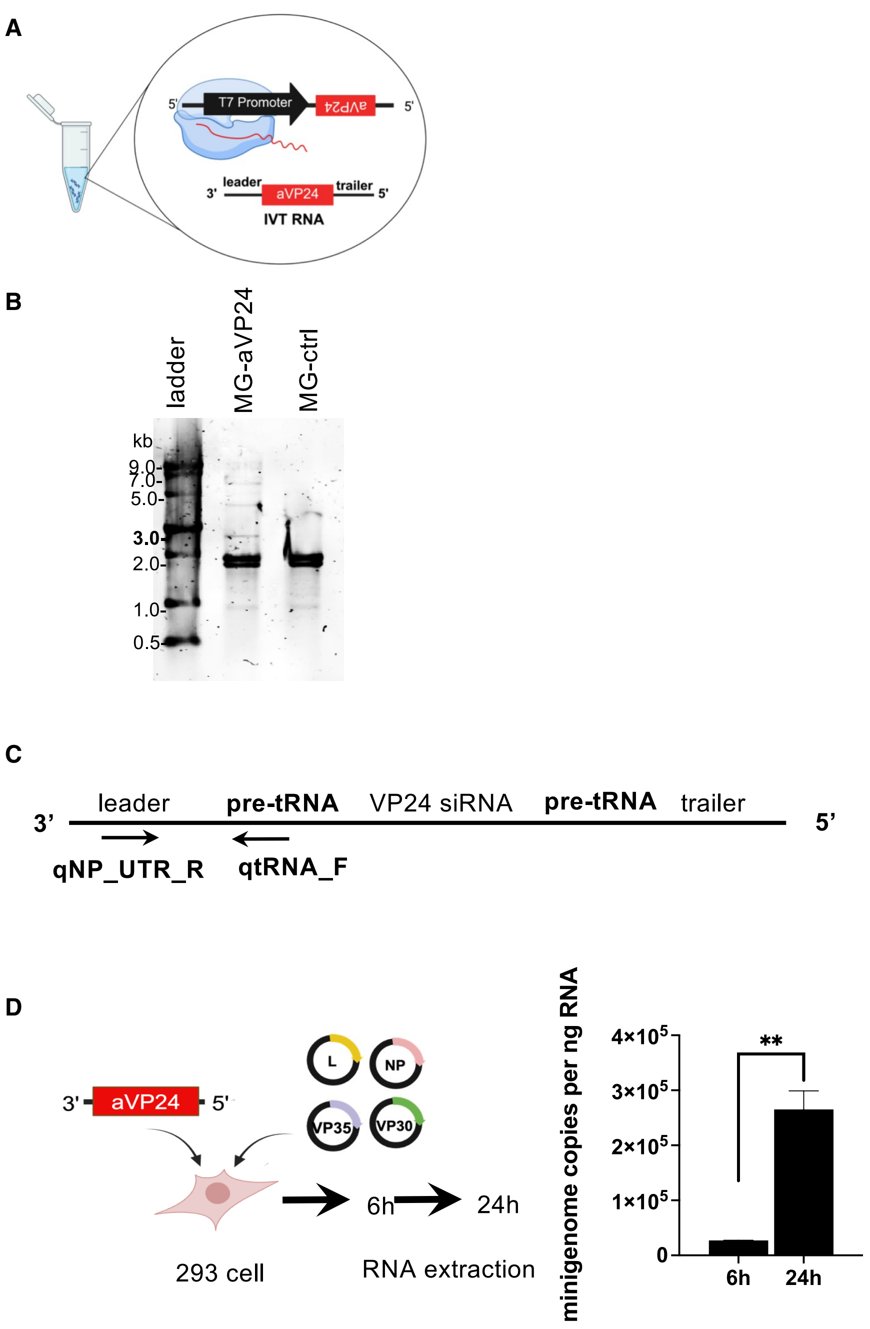

Figure 3. IVT 1*cis*-MG-anti-VP24 RNA

(A) *In vitro* transcription (IVT) of MG. (B) Analysis of the integrity and size of IVT therapeutic MG RNA and control MG RNA using agarose gel electrophoresis. (C) Overview of the 1*cis*-MG-anti-VP24 vRNA and qPCR primer binding sites. (D) Scheme and results of IVT 1*cis*-MG-anti-VP24 RNA amplification in the presence of EBOV helper proteins. Quantification of 1*cis*-MG is measured by RT-qPCR targeting the payload region using primer set (qNP_UTR R:5′-GCTTGGGGTAAAACATTGG-3′; qtRNA-F: 5′-ACTTGAACCCTGGACCCTCA-3′). Data represent mean ± S.D (n = 3 independent biological replicates). Paired t test was used for statistical analysis: $^{**}p \leq 0.01$.

Therapeutic MG-encoding anti-VP24 shRNA efficiently inhibits surrogate virus genome replication, transcription, and production of infectious particles

To assess the antiviral effect of 1*cis*-MG-anti-VP24 RNA, we utilized an EBOV tetracistronic-MG (4*cis*-MG) system as a surrogate virus.[43] The 4*cis*-MG encodes three EBOV proteins VP40, GP, and VP24, as well as a nanoLuciferase (nLuc) reporter.[43,44] This design enables modeling of the EBOV life cycle, including genome replication, transcription, packaging, and the production of infectious trVLPs, when RNP proteins are provided *in trans* (Figure 4A). While infectious EBOV is required to be handled in biosafety level 4 (BSL-4) laboratories due to its virulence, 4*cis*-MG can be safely handled under BSL-2 conditions.

Using this system, we first evaluated the VP24 silencing effects of 1*cis*-anti-VP24 by quantifying levels of VP24 mRNA expressed from 4*cis*-MG. The 1*cis*-anti-VP24 or empty MG was co-transfected in producer cells (passage 0; P0) with 4*cis*-MG and helper plasmids (Figure 4A). Under these conditions, a significant reduction in VP24 mRNA levels was observed in the presence of 1*cis*-MG-anti-VP24 RNA compared with the 1*cis*-MG-empty control (Figure 4B, left). Although to a lesser extent than the therapeutic MG, the 1*cis*-MG-empty control also exhibited an inhibitory effect relative to the L^+ condition. Here, the cells were transfected with 4*cis*-MG and all helpers but not the 1*cis*-MG; the observed inhibition is likely due to competitive replication with the surrogate virus genome (Figure 4B, left). A similar trend was also observed in 4*cis*-MG viral RNA (vRNA) levels when using the same qPCR primer set targeting the VP24 region, showing a significant reduction in the presence of 1*cis*-anti-VP24 compared to the empty MG control (Figure 4B, right). These results strongly suggest that 1*cis*-anti-VP24 can effectively silence not only VP24 mRNA, but also 4*cis*-MG vRNA by targeting the VP24 sequence on the cRNA.

To assess whether the VP24 silencing effect negatively impacts the packaging of 4*cis*-MG into infectious trVLPs, supernatants from the previous P0 cells were transferred to target cells (passage 1; P1) (Figure 4A), and nLuc reporter signals in P1 cells were subsequently analyzed (Figure 4C). We observed a significant reduction of over

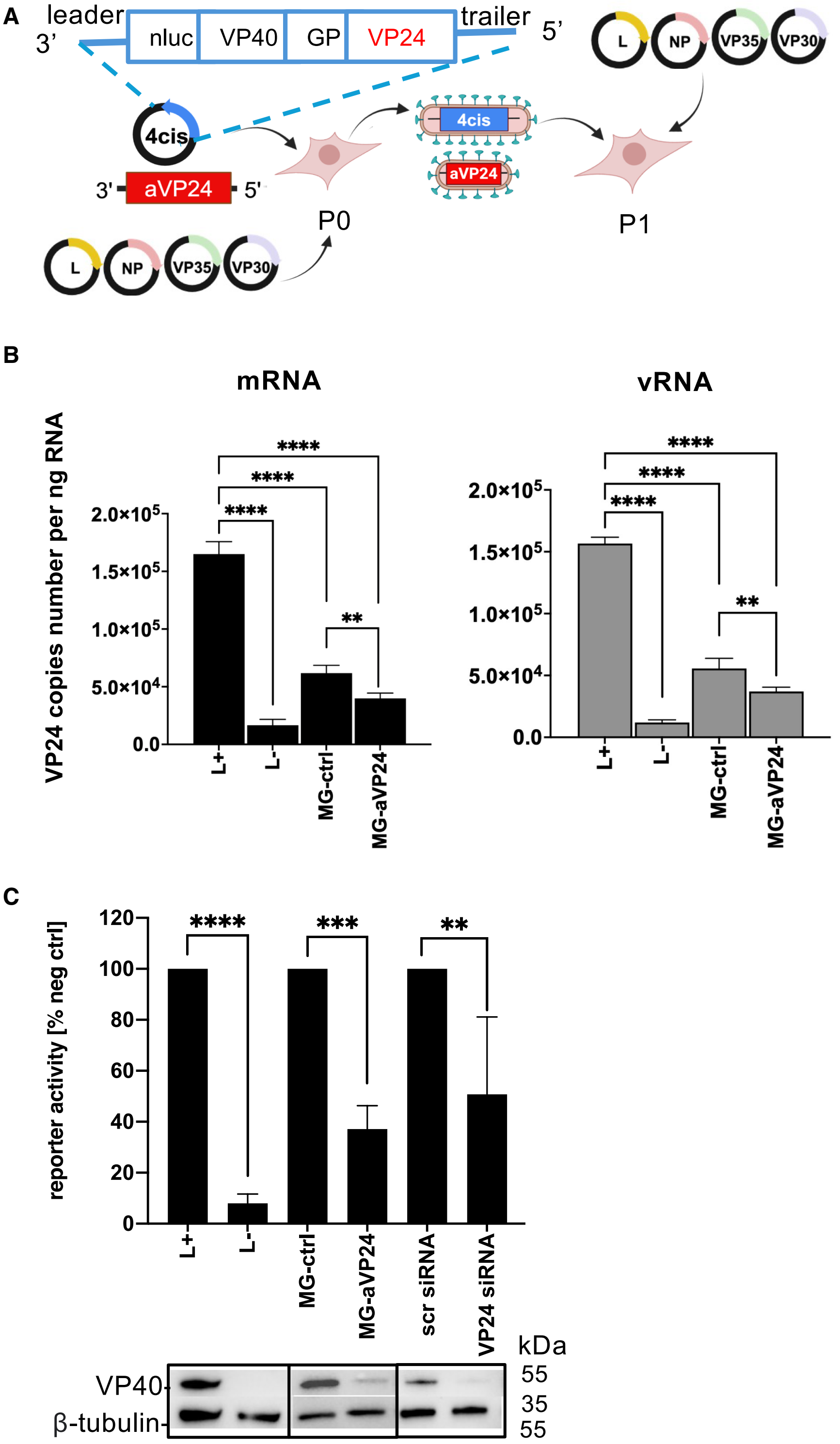

Figure 4. 1*cis*-MG-anti-VP24 inhibits the replication and transcription of 4*cis*-MG

(A) Illustration of 4*cis*-MG cassette and co-transfection of IVT 1*cis*-MG-anti-VP24 RNA, 4*cis*-MG and helper plasmids (pCAGGS-L, VP30, VP35 and NP) into 293 cells. (B) Two-step RT RT-qPCR was performed for quantification of VP24 vRNA level RT using vRNA-specific primer and VP24 mRNA-level RT with Oligo d(T) in P0 cells. Copy numbers were calculated based on a standard curve generated using serially diluted 4*cis*-MG plasmid DNA and normalized to copies per ng RNA. (C) nLuc reporter activity and viral protein expression in helper plasmid pre-transfected P1 target cells. L$^+$: cells transfected with helper plasmids. L$^-$: cells transfected with helper plasmids except for EBOV L polymerase-expressing plasmid. An empty pCAGGS was used to account for total plasmid deficit. Bar graph data represent mean ± S.D (n = 3 independent biological replicates) are shown as percentage reduction of luciferase activity relative to individual negative control, which was set at 100%. Western blots shown are using lysate combined from triplicate wells. $^{**}p \leq 0.01$, $^{***}p \leq 0.001$, $^{****}p \leq 0.0001$; ordinary one-way ANOVA was used for statistical analysis.

60% in the nLuc reporter signal with 1*cis*-anti-VP24 RNA transfection when compared with the control MG (Figure 4C). Similarly, transfection of 25 nM synthesized VP24 siRNA into P0 cells, used as a positive control, reduced nLuc activity by approximately 50% compared with the scrambled siRNA. The luciferase activity in L-absent (L$^-$) condition relative to L$^+$ was less than 10%.

In addition to the nLuc reporter signal, the expression level of VP40—one of the EBOV proteins encoded by the 4*cis*-MG—in P1 cells was evaluated. Consistent with nLuc activity, VP40 levels were reduced in both 1*cis*-MG-anti-VP24 and synthetic VP24 siRNA transfections when compared to their respective controls (Figure 4C). The level of VP40 expression under the L$^-$ condition was below the detection limit, as expected. Collectively, these findings suggest that 1*cis*-MG-anti-VP24 inhibits the production of infectious 4*cis*-MG trVLPs through its VP24 silencing effect, thereby effectively suppressing replication of this surrogate virus.

Development of a therapeutic MG encoding a host antiviral protein-derived peptide

Previous studies have shown that the host protein RBBP6 inhibits EBOV RNA synthesis by disrupting the interaction between EBOV NP and VP30 proteins.[45,46] These studies also identified a 23-amino-acid peptide from RBBP6 (RBBP6$_{549-571}$) as a mediator of this inhibitory effect. Given this, RBBP6$_{549-571}$ was selected as an example of using host proteins as a payload in the therapeutic MG.

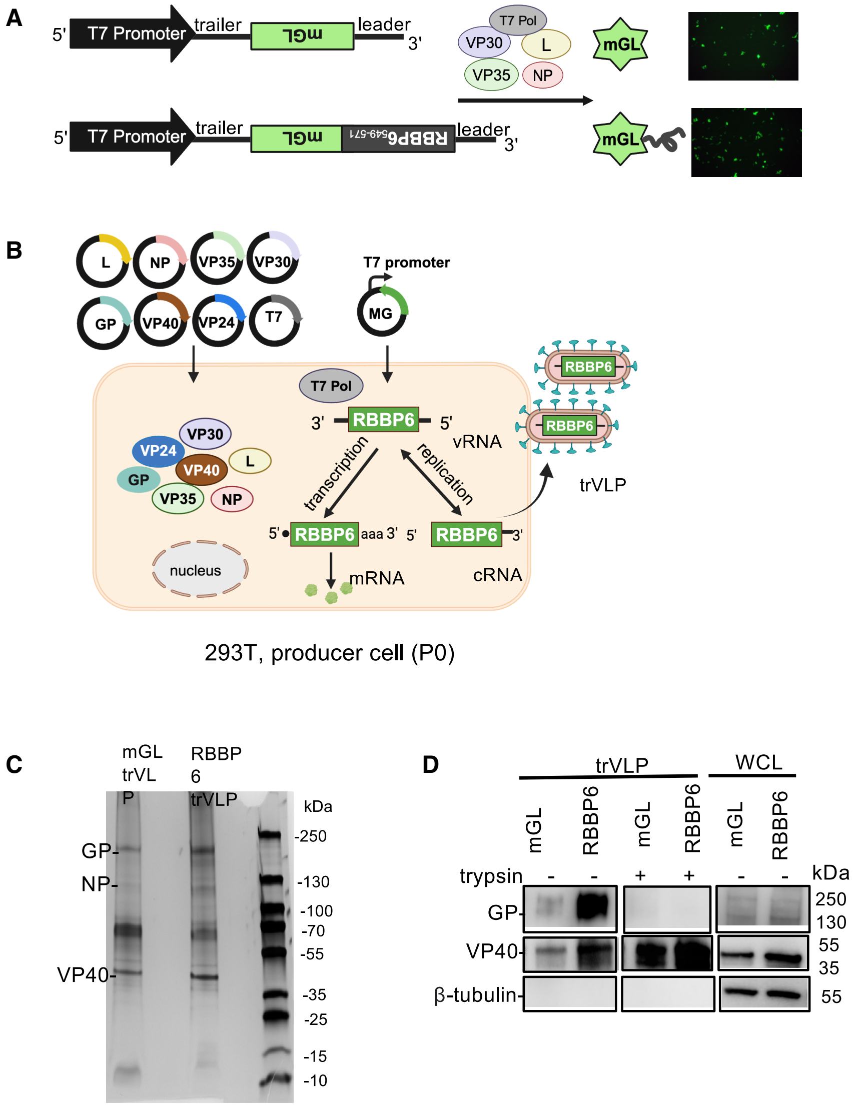

Figure 5. Production and analysis of 1*cis*-MG trVLP

(A) Diagram of 1*cis*-MG-mGL and 1*cis*-MG-RBBP6$_{549-571}$-mGL plasmids, and fluorescence images showing mGreenlantern expression in cells co-transfected with plasmids expressing 1*cis*-MG, EBOV helpers, and T7 polymerase. (B) A schematic illustration of the 1*cis*-MG-RBBP6 trVLP production. 293T cells are transfected with expression plasmids for all seven viral proteins, including RNP proteins NP, VP35, VP30, and L, and proteins VP40, GP, and VP24 that involved in particle budding/packaging/entry, as well as a 1*cis*-MG and the accessory T7 RNA polymerase (T7) for initial transcription of MG plasmid. The vRNA is replicated through a cRNA intermediated by the viral proteins NP, VP35, and L and transcribed by these proteins and VP30 into mRNAs encoding RBBP6 peptide. vRNA minigenomes are packaged and bud as trVLPs. (C) Silver staining of purified 1*cis*-MG-mGL and 1*cis*-MG-RBBP6 trVLPs. (D) Both trVLPs either treated with TPCK trypsin (0.1 μg/mL) or not and producer cell whole cell lysate (WCL) were analyzed for presence of GP, VP40 and β-tubulin with western blotting. Western blot images are cropped to focus on the target bands of interest.

RBBP6$_{549-571}$ was fused to the green fluorescent protein mGreenLantern (mGL) at the N-terminus and cloned into the 1*cis*-MG (1*cis*-MG-RBBP6$_{549-571}$–mGL). This construct was compared to a 1*cis*-MG-mGL lacking the RBBP6 peptide in the subsequent assays (Figure 5A). When these MGs were transfected into P0 cells, similar levels of mGL signals were observed in cells either co-transfected (Figure 5A) or up to 24 h prior to transfection of EBOV RNP plasmids (Figure S1B).

To assess the potency of trVLPs for therapeutic MG delivery, trVLPs containing 1*cis*-MG-RBBP6$_{549-571}$-mGL or 1*cis*-MG-mGL were then generated and thoroughly characterized. trVLPs were produced referring to previously established methods by transfecting 1*cis*-MG-RBBP6$_{549-571}$–mGL or 1*cis*-MG-mGL plasmid along with plasmids for all seven EBOV proteins (Figure 5B).[33] trVLP production was quantified for protein content by bicinchoninic acid (BCA) assay (data not shown). Silver staining and western blot analyses confirmed the presence of GP and VP40 in both trVLP preparations (Figures 5C and 5D). Protease protection assay demonstrated complete digestion of GP following trypsin treatment, while VP40 remained intact, confirming the integrity of the viral particles with the presence of GP displayed on their surface (Figure 5D).

Therapeutic MG RBBP6$_{549-571}$ trVLPs inhibit surrogate virus genome replication, transcription, and production of infectious particles

The therapeutic effect of 1*cis*-MG-RBBP6$_{549-571}$-mGL, delivered via trVLPs, was ultimately evaluated using the EBOV 4*cis*-MG surrogate virus (Figure 6A). 293 cells were transfected with plasmids expressing 4*cis*-MG and helper proteins and subsequently treated with therapeutic trVLPs (P1). We observed a more than 50% reduction in nLuc activity with 1*cis*-MG-RBBP6$_{549-571}$-mGL trVLPs when compared to 1*cis*-MG-mGL trVLPs (Figure 6B). The supernatants from these P1 cells were then passaged onto fresh cells expressing helper proteins three times (from P2 to P4) (Figure 6A). Notably, the inhibitory effect of 1*cis*-MG-RBBP6$_{549-571}$-mGL on 4*cis*-MG replication became more pronounced with increased passages, demonstrating reductions in nLuc activity of approximately 60% in P2, 80% in P3, and 90% in P4, when compared to the negative control 1*cis*-MG-mGL trVLPs (Figure 6B). Consistent with these observations, a concurrent decrease in the expression of VP40, encoded by 4*cis*-MG, was also detected in each passage (Figure 6C), further supporting the inhibition of 1*cis*-MG-RBBP6$_{549-571}$-mGL on 4*cis*-MG surrogate virus.

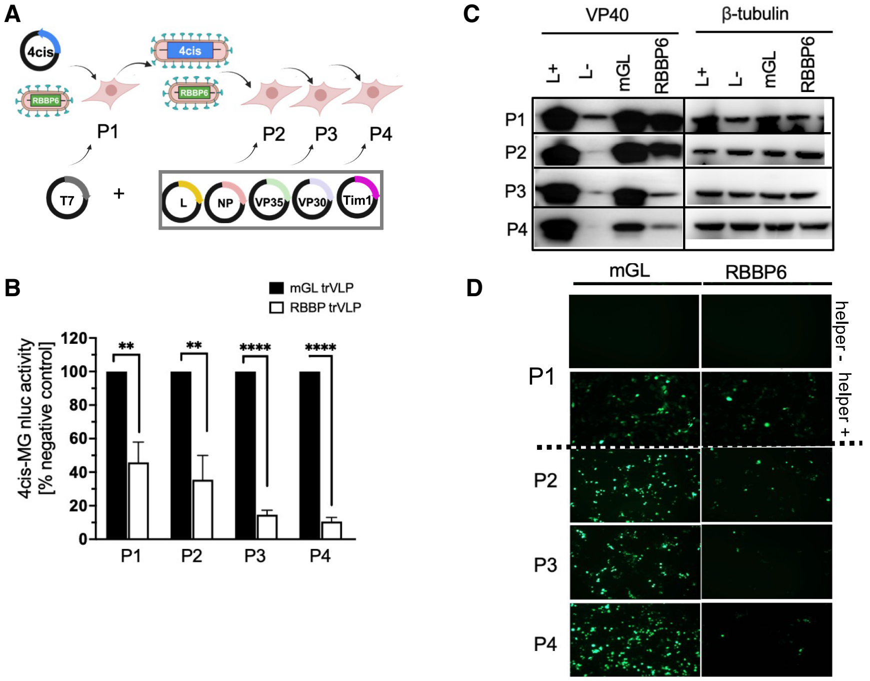

Figure 6. 1*cis*-MG-RBBP6 trVLP inhibits the expression of 4*cis*-MG

(A) Illustration of 1*cis*-MG-RBBP6 trVLP infection in 293 cells pre-transfected with 4*cis*-MG, helper plasmids and Tim1 plasmid. Supernatant containing trVLPS with 1*cis*-MG-RBBP6 or 4*cis*-MG from P1 cells was passaged 72 h after infection onto P2 cells that pre-transfected with expression plasmids encoding the EBOV helper proteins, as well as Tim1. Supernatant of each infection was continuously passaged every 72 h for a total of 4 passages. The means and standard deviations from 3 independent experiments are shown. (B) Reporter activity in target cells pre-transfected with helper and Tim1 expressing plasmid. The nLuc signals in control 1*cis*-MG-mGL trVLP-infected cells at each passage were set as 100%. The means and SD from 3 independent experiments were plotted. (C) Expression levels of viral protein VP40 and β-tubulin in cells from each passage. (D) Fluorescence imaging of cells with original magnification of ×250 from each passage, including P1, without helper plasmids pre-transfected. Unpaired *t* test was used for statistical analysis: $^{*}p < 0.05$, $^{**}p < 0.01$, $^{***}p < 0.001$, $^{****}p < 0.0001$.

Therapeutic MG or control MG are only expressed when helper proteins are provided *in trans* (Figure 6D). Importantly, the negative control trVLPs continued to amplify across passages, evidenced by an increase in mGL signals by passage 4 (Figure 6D). This indicates that the control 1*cis*-MG was able to utilize viral proteins provided by helper plasmids and 4*cis*-MG to replicate itself. In contrast, trVLPs containing 1*cis*-MG-RBBP6$_{549\text{-}571}$-mGL showed a progressive decline in mGL signals over several passages (Figure 6D) in parallel to a decreased replication of 4*cis*-MG (Figures 6B and 6C). Overall, these results provide strong support for the concept of the therapeutic MG as an infection-sensing mechanism and highlight its safety and potency.

DISCUSSION

In this study, we tested a novel nucleic acid antiviral therapy that exploits viral replication machinery to drive therapeutic expression. The results here demonstrate proof-of-concept that therapeutic MGs encoding anti-viral shRNAs or proteins can antagonize EBOV surrogate genome replication and progeny virus production.

This therapeutic MG approach achieves specificity by utilizing elements derived from the negative-sense viral RNA genome, in this case from EBOV. Unlike positive-sense viral RNA genomes, which act as mRNA and are immediately translated by the host ribosomes upon cell entry, negative-sense viral RNA genomes must first be transcribed into positive-sense RNA by viral proteins before translation can occur. Consequently, therapeutic transgenes encoded on a negative-sense RNA MG are not transcribed or translated in uninfected cells and thus remain “silent.” In the absence of a subsequent EBOV infection, the therapeutic RNA will be naturally degraded over time. These features may enhance safety by minimizing off-target effects—a common limitation of small-molecule drugs that lack specificity. Beyond ensuring stringent restriction of therapeutic expression to infected cells, this platform also enables the cessation of therapeutic expression as the virus is cleared, as demonstrated in our results using a surrogate virus (Figure 6D). This self-limiting nature of the therapeutic MG potentially synchronizes the level and kinetics of therapy with the kinetics of the viral infection.

We propose that the inhibitory effect of therapeutic MGs arises from two key antiviral actions: competitive replication and therapeutic amplification. Therapeutic MGs retain viral elements for genome replication and transcription as well as genome packaging into virions, enabling them to compete for resources with the wild-type virus for viral proteins. Supporting this, the control MG—lacking a therapeutic transgene—also exhibited some antiviral effects against the surrogate virus, albeit to a lesser extent than the therapeutic MG (Figure 4B). Importantly, previous studies have shown that smaller genomes replicate more efficiently than their full-length counterparts.[47–52] Our designed therapeutic MGs are significantly smaller than full-length EBOV genome. This not only enhances competitive MG replication, but also ensures robust amplification of the therapeutic payload, further contributing to their antiviral potency. Additionally, the ability of the therapeutic MG to be packaged into virions strengthens its amplifiable nature by facilitating the delivery of MGs to additional target cells.

Aside from EBOV, Sudan virus and Marburg virus (MARV) are additional filoviruses which have also caused several devastating outbreaks in sub-Saharan Africa in recent years.[53–56] Many filovirus

proteins are compatible, and their MGs can be supported by nucleocapsid proteins from other filovirus species.[57–59] Importantly, two transgenes we have tested in this study—VP24 shRNA and RBBP6 peptide—demonstrate broad antiviral potential across all filoviruses. VP24 has been identified as an interferon antagonist in all ebolavirus species,[33,60–63] and silencing VP24 in MARV-infected cells significantly inhibits budding of progeny virions, albeit viral transcription and replication were unimpaired.[34,64] The anti-filovirus effect of the $RBBP6_{549\text{-}571}$ peptide is also promising, as its target, the PPxPxY motif in viral NP, is conserved among all filoviruses. These insights strongly suggest that VP24 siRNA and the $RBBP6_{549\text{-}571}$ peptide could serve as effective pan-filovirus therapeutics.[45,46,65] It is also worth noting that promoter and packaging signals have been identified not only for EBOV but also for other filoviruses.[30] Thus, the therapeutic MGs developed in this study can be easily adapted for targeting different filoviruses by swapping the signal sequences, offering a potential new approach for pan-filovirus antiviral treatments during outbreaks.

We are aware that the antiviral effect of therapeutic 1*cis*-MGs has only been tested in the surrogate model (4*cis*-MG) under BSL-2 conditions, which we currently have access to. Testing with infectious viruses in high-containment facilities will enable us to more comprehensively characterize the MG therapeutic platform.

While this study has primarily demonstrated proof-of-concept for the platform, future research should also expand on evaluating the *in vivo* therapeutic potency of the MGs to further establish their clinical potential. One promising approach for therapeutic MG RNA delivery *in vivo* is the use of lipid nanoparticles (LNPs), which have been widely utilized as vehicles for delivering nucleic acids.[35,66–72] Previous studies have demonstrated a significant portion of LNPs administrated to animals targets the liver,[73–75] which may be advantageous for targeting filoviruses, as the liver is the primary target organ for filovirus replication.[76–82] Alternatively, the therapeutic MG RNA could be delivered *in vivo* using EBOV trVLPs or, in the case of other RNA viruses such as VSV, by incorporating self-cleaving ribozymes or *trans*-cleaving pre-tRNA sequences flanking the negative-sense therapeutic MG into their genomes. Incorporating the therapeutic MG into viral particles displaying EBOV GP on their surface may extend the functionality of this antiviral platform to additionally serve as a therapeutic vaccine.[83–87]

Furthermore, our therapeutic approach could also be extended to provide more long-lasting or perhaps permanent antiviral protection by delivering the negative-strand MG not as RNA, but expressing from a DNA-based vector, such as an adeno-associated virus (AAV) vector, driven by RNA polymerase II (Pol II) or RNA polymerase III (Pol III) promoter.[88] Future studies will focus on evaluating the delivery efficiency, antiviral effects, and host immune responses in infected animal models treated with the therapeutic MGs.

Overall, the therapeutic MGs presented in this study represent a novel and promising platform for antiviral therapy, combining robust potency with a distinct safety profile. This platform leverages viral proteins from ongoing infections to amplify therapeutic genes specifically within those cells. While this study focused on two therapeutic genes that directly target the virus life cycle, the repertoire of therapeutic transgene can be expanded to include various other targets, such as host pathogenic responses. In addition, the efficacy of these negative-strand MG therapies to attenuate EBOV provides proof-of-concept for potential applications against other RNA viruses, both negative- and positive-strand, as well as DNA viruses, by delivering appropriate antisense RNAs or cDNAs. The versatility of this therapeutic MG platform to target a wide range of viruses and accommodate diverse payloads will significantly advance our ability to combat a variety of infectious viral diseases.

MATERIALS AND METHODS

Plasmids and RNA

The therapeutic expression MG plasmids 1*cis*-MG-anti-VP24, 1*cis*-MG-$RBBP6_{549\text{-}571}$-mGL, and their negative controls, 1*cis*-MG-empty and 1*cis*-MG-mGL, were constructed using a T7 polymerase-driven EBOV monocistronic MG plasmid backbone. Briefly, the synthesized transgenes mGL, $RBBP6_{549\text{-}571}$-mGL, and anti-VP24 shRNA (GenScript Biotech, Piscataway, NJ) were individually cloned into BsmBI-v2 (New England Biolabs, Ipswich, MA)-linearized pATX-T7-1*cis*-EBOVMG-nLuc using In-Fusion cloning kit (Takara Bio, Shiga, Japan). The 1*cis*-MG-empty construct was created by removing the nLuc reporter gene.

The T7 polymerase-driven 4*cis*-MG plasmid, encoding EBOV proteins VP40, GP, VP24, and nLuc luciferase, was previously constructed.[44]

Expression plasmids for EBOV proteins and T7 polymerase in pCAGGs vectors have been previously described.[33,43]

The psiCHECK2-VP24 plasmid was created by inserting the VP24 full-length sequence downstream of the *Renilla* luciferase gene in the psiCHECK2 plasmid (Promega, Madison, WI).

1*cis*-MG RNA was synthesized via IVT using T7 RNA polymerase with the HiScribe T7 High Yield RNA Synthesis Kit (New England Biolabs, Ipswich, MA). Therapeutic MG plasmids and their respective negative controls, linearized with MluI and NotI, were used as templates for synthesizing IVT RNAs.

Cell culture

293 (American Type Culture Collection [ATCC]; CRL-1573) and 293T (ATCC, CRL-3216) cells were grown in Dulbecco's Modified Eagle Medium (DMEM), supplemented with 10% fetal bovine serum (FBS), 100 U/mL penicillin, and 100 mg/mL streptomycin. All cells were incubated at 37°C in a humidified atmosphere equilibrated to 5% CO_2.

Transfection and infection

Unless otherwise specified, all transfection reagents were purchased from Mirus Bio (Madison, WI). TransIT-LT1 was used for DNA

transfection, TransIT-mRNA was used for RNA transfection, and TransIT-X2 was used for siRNA or siRNA mixed with DNA transfection, following the manufacturer's instructions. All transfection complexes were incubated in Opti-MEM (Gibco) at room temperature (~22°C) for the recommended duration.

Before infecting 293 cells grown in a 24-well plate with the trVLP-containing supernatant, the culturing medium was removed, and the cells were gently washed with PBS. Then, 100 μL of supernatant was added on top and incubated in a 37°C incubator with 5% CO_2 for 1 h, gently rocking the plate every 15 min. After incubation, 500 μL of DMEM culturing media was added to replace the supernatant.

psiCHECK-2 assay

On the day prior to transfection, 100 μL of 293 cells (1 × 10^4 cells) were added to a 96-well plate. The cells were co-transfected with 1*cis*-MG-anti-VP24 and helper plasmids or synthesized siRNAs along with psiCHECK2-VP24 or psiCHECK2. Forty-eight h post transfection, the cells were lysed, and a dual-luciferase reporter assay (Promega, Madison, WI) was performed to measure *Renilla* luciferase and internal control firefly luciferase signals. The ratio of *Renilla* to firefly luciferase signals was normalized to the respective negative control, which was assigned a value of 100%, and expressed as a percentage of gene expression.

MG assay

293 cells were seeded at a density of 8 × 10^4 cells per well in 24-well plates 24 h before transfection. Cells (passage 0; P0 cells) were transfected with plasmids: 200 ng of pCAGGS-L, 25 ng each of pCAGGS-NP and pCAGGS-VP35, 15 ng of pCAGGS-VP30, 50 ng of pCAGGS-T7p, 100 ng of EBOV 4*cis*-MG-nluc, and 10 ng of internal control pGL4.10[luc2] (Promega, Madison, WI). Therapeutic plasmids or RNAs were added 6–8 h after transfection. At 72 h post-transfection, supernatants containing trVLPs were harvested and centrifuged at 500 × g for 5 min to remove cell debris. 100 μL of cleared supernatants were used to infect 293 cells (passage 1; P1 cells), which had been pre-transfected for 24 h with the same amount of helper plasmids as used for P0. Both P0 and P1 cells were lysed in 100 μL of 1× passive lysis buffer (Promega, Madison, WI) after 72 h of transfection or infection. 50 μL of the lysate was used to evaluate nLuc luciferase expression using the Nano-Glo dual-luciferase reporter assay (Promega, Madison, WI) according to the manufacturer's instructions. The remaining cell lysate were saved for subsequent western blot analysis.

Western blot

Whole cell lysates were harvested after the removal of media and a single wash with 1× PBS. Cells were extracted in 1× passive lysis buffer supplemented with Protease Inhibitor Cocktail (Sigma-Aldrich, Burlington, MA). Samples were incubated for 10 min at room temperature (~22°C) with rotation, followed by centrifugation at 16,000 × g at 4°C for 10 min to remove cell debris. Equal volumes of samples were mixed with 2× Laemmli sample buffer (Bio-Rad, Hercules, CA) containing 10 mM DTT and boiled on a heat block for 10 min before loading onto SDS-PAGE gels. Proteins were transferred to a PVDF membrane using a semi-dry transfer method at 15V for 45 min (Bio-Rad, Hercules, CA). Nonspecific binding to the membranes was blocked with 5% (w/v) skim milk powder solutions in TBS-Tween. Blots were probed overnight at 4°C with anti-VP40 (clone 5B12, IBT BioServices, cat. no. 0201-017) and anti-VP24 (Polyclonal, SinoBiological, cat. no.40454-T46). Afterward, the membranes were washed three times for 10 min each with TBS-Tween buffer, followed by incubation with HRP-conjugated secondary antibodies. Western blot signals were detected using chemiluminescence by adding SuperSignal West Femto substrate (Thermo Fisher Scientific, Waltham, MA) and imaged using the Bio-Rad ChemiDoc imaging system. After imaging, the membranes were probed with anti-β-tubulin (polyclonal, Abcam, cat. no. ab6046) for 2 h at room temperature (~22°C) as a loading control.

RT-qPCR for mRNA and vRNA

The therapeutic effects of 1*cis*-MG-anti-VP24 and 1*cis*-MG-RBBP$6_{549-571}$-mGL on 4*cis*-MG were examined by quantifying 4*cis*-MG RNA in transfected 293 cells. Cells in 24-well plates were lysed in 500 μL TRIzol reagent for total RNA extraction at 6-, 24-, and 72 h post-transfection using Direct-zol RNA Miniprep Kits (Zymo Research, Irvine, CA). The extracted RNA was eluted in 100 μL RNase-free water and quantified using a NanodropOne (ThermoFisher). 200 ng of each isolated RNA was reverse transcribed (RT) with SuperScript IV Reverse Transcriptase (Thermo Fisher Scientific, Waltham, MA) for mRNA and vRNA using oligo d(T) and strand-specific primers, respectively, resulting in a 20 μL reaction volume. The resulting cDNAs were diluted in 180 μL nuclease-free water. For the qPCR reaction, 8 μL diluted cDNA was used as the template, mixed with 10 μL SYBR green master mix (Applied Biosystems, Waltham, MA) and 2 μL paired primer (500 nM). The qPCR reaction was loaded into a standard 96-well PCR plate and spun at 150 × g for 1 min before running on the QuantStudio 3 Real-time PCR system (Applied Biosystems, Waltham, MA). 4*cis*-MG plasmid DNA was used to standardize genome copy numbers, expressed as "viral genome copies." Genome copy numbers for each sample were determined by automatic analysis of threshold cycle (Ct) values with the QuantStudio 3 software and calculated based on the measured total extracted RNA amount. Results were expressed as "viral genome copies per ng RNA."

trVLPs

To produce therapeutic 1*cis*-MG containing trVLPs, 293T producer cells (P0) were seeded at a density of 1 × 10^6 cells in 2.5 mL of DMEM supplemented with 10% FBS and 1% pen/strep per well of 6-well plates. A ratio of 1 μg DNA to 3 μL of TransIT LTI (Mirus Bio, Madison, WI, USA) was used for transfecting the cells with 4 μg of pCAGGS-L, 1 μg of pCAGGS-GP, 0.5 μg of pCAGGS-NP, 1 μg of pCAGGS-VP40, 0.5 μg of pCAGGS-VP35, 0.3 μg of pCAGGS-VP30, 0.03 μg of pCAGGS-VP24, 1 μg of pCAGGS-T7, and 1 μg of 1*cis*-MG. The media was changed to DMEM with 10% FBS and 1% pen/strep 24 h post transfection.

Seventy-two h post-transfection, 7.5 mL of supernatants from three wells were collected and centrifuged for 5 min at 150 × g in a benchtop centrifuge to remove cell debris. 7 mL of clarified supernatants were then applied to a 3 mL 20% sucrose layer in NTE buffer (10mM Tris, 1 mM EDTA, 100 mM NaCl) in a Beckman centrifuge tube (cat. no. C14293) and centrifuged at 32,000 rpm in an SW32-TI rotor for 2 h at 4°C using a Beckman L7 ultracentrifuge. Pellets were resuspended in 50 μL of NTE buffer, and total protein concentrations were determined using BCA assay (Pierce BCA Protein Assay Kits, Thermo Fisher Scientific, cat. no. 23225). Silver staining (Pierce Silver Stain Kit, Thermo Fisher Scientific, cat. no. 24612) and western blot were performed to assess individual viral proteins. P0 transfected cells used to generate the trVLPs were lysed in 1xPassive lysis buffer and analyzed by western blot.

Statistical analyses

All experiments were performed as at least three independent biological replicates. Statistical analyses were performed using *t* test or one-way ANOVA with GraphPad Prism 10 v.10.0.2.

DATA AVAILABILITY

All data generated from this study are available within the article.

ACKNOWLEDGMENTS

This project was supported by Mayo Clinic's Foundation for Medical Education and Research and the National Institute of Allergy and Infectious Diseases (R01 AI134937-01A1). B.J.P. was supported in part by the T32 training grant 5T32AI132165. The opinions, interpretations, conclusions, and recommendations are those of the authors and are not necessarily endorsed by Mayo Clinic or the NIH.

AUTHOR CONTRIBUTIONS

Conceptualization, S.Y. and L.W.; methodology, L.W., B.N.Z., B.J.P., and S.Y.; software, L.W.; validation, L.W.; formal analysis, L.W.; investigation, L.W. and S.Y.; resources, all authors; data curation, L.W.; writing – original draft preparation, L.W.; writing – review & editing, all authors; visualization, L.W.; project administration, L.W.; supervision, S.Y. and M.A.B.; funding acquisition, M.A.B. All authors have read and agreed to the published version of the manuscript.

DECLARATION OF INTERESTS

This work was filed in the United States Patent and Trademark Office on June 2, 2025, with application # PCT/US25/31903, titled "Transcription and replication particles and their use as antiviral therapies."

SUPPLEMENTAL INFORMATION

Supplemental information can be found online at https://doi.org/10.1016/j.omtn.2025.102722.

REFERENCES

1. Baize, S., Pannetier, D., Oestereich, L., Rieger, T., Koivogui, L., Magassouba, N., Soropogui, B., Sow, M.S., Keïta, S., De Clerck, H., et al. (2014). Emergence of Zaire Ebola virus disease in Guinea. N. Engl. J. Med. *371*, 1418–1425. https://doi.org/10.1056/NEJMoa1404505.
2. Petti, S., Messano, G.A., Vingolo, E.M., Marsella, L.T., and Scully, C. (2015). The face of Ebola: changing frequency of haemorrhage in the West African compared with Eastern-Central African outbreaks. BMC Infect. Dis. *15*, 564. https://doi.org/10.1186/s12879-015-1302-4.
3. Lu, H.J., Qian, J., Kargbo, D., Zhang, X.G., Yang, F., Hu, Y., Sun, Y., Cao, Y.X., Deng, Y.Q., Su, H.X., et al. (2015). Ebola Virus Outbreak Investigation, Sierra Leone, September 28-November 11, 2014. Emerg. Infect. Dis. *21*, 1921–1927. https://doi.org/10.3201/eid2111.150582.
4. Ohuabunwo, C., Ameh, C., Oduyebo, O., Ahumibe, A., Mutiu, B., Olayinka, A., Gbadamosi, W., Garcia, E., Nanclares, C., Famiyesin, W., et al. (2016). Clinical profile and containment of the Ebola virus disease outbreak in two large West African cities, Nigeria, July-September 2014. Int. J. Infect. Dis. *53*, 23–29. https://doi.org/10.1016/j.ijid.2016.08.011.
5. Schieffelin, J.S., Shaffer, J.G., Goba, A., Gbakie, M., Gire, S.K., Colubri, A., Sealfon, R.S.G., Kanneh, L., Moigboi, A., Momoh, M., et al. (2014). Clinical illness and outcomes in patients with Ebola in Sierra Leone. N. Engl. J. Med. *371*, 2092–2100. https://doi.org/10.1056/NEJMoa1411680.
6. Haaskjold, Y.L., Bolkan, H.A., Krogh, K.Ø., Jongopi, J., Lundeby, K.M., Mellesmo, S., Garcés, P.S.J., Jøsendal, O., Øpstad, Å., Svensen, E., et al. (2016). Clinical Features of and Risk Factors for Fatal Ebola Virus Disease, Moyamba District, Sierra Leone, December 2014-February 2015. Emerg. Infect. Dis. *22*, 1537–1544. https://doi.org/10.3201/eid2209.151621.
7. Nsio, J., Ardiet, D.L., Coulborn, R.M., Grellety, E., Albela, M., Grandesso, F., Kitenge, R., Ngwanga, D.L., Matady, B., Manangama, G., et al. (2023). Differential symptomology of possible and confirmed Ebola virus disease infection in the Democratic Republic of the Congo: a retrospective cohort study. Lancet Infect. Dis. *23*, 91–102.
8. Sterk, E. (2008). Filovirus Haemorrhagic Fever Guideline (Medecins Sans Frontieres).
9. WHO. Ebola outbreak 2014-2016 - West Africa. https://www.who.int/emergencies/situations/ebola-outbreak-2014-2016-West-Africa.
10. Hoenen, T., Groseth, A., and Feldmann, H. (2019). Therapeutic strategies to target the Ebola virus life cycle. Nat. Rev. Microbiol. *17*, 593–606. https://doi.org/10.1038/s41579-019-0233-2.
11. Lei, S., Chen, X., Wu, J., Duan, X., and Men, K. (2022). Small molecules in the treatment of COVID-19. Signal Transduct. Target. Ther. *7*, 387. https://doi.org/10.1038/s41392-022-01249-8.
12. Lu, L., Su, S., Yang, H., and Jiang, S. (2021). Antivirals with common targets against highly pathogenic viruses. Cell *184*, 1604–1620. https://doi.org/10.1016/j.cell.2021.02.013.
13. Edwards, M.R., and Basler, C.F. (2019). Current status of small molecule drug development for Ebola virus and other filoviruses. Curr. Opin. Virol. *35*, 42–56. https://doi.org/10.1016/j.coviro.2019.03.001.
14. Bai, J.P.F., and Abernethy, D.R. (2013). Systems pharmacology to predict drug toxicity: integration across levels of biological organization. Annu. Rev. Pharmacol. Toxicol. *53*, 451–473. https://doi.org/10.1146/annurev-pharmtox-011112-140248.
15. Furuta, Y., Gowen, B.B., Takahashi, K., Shiraki, K., Smee, D.F., and Barnard, D.L. (2013). Favipiravir (T-705), a novel viral RNA polymerase inhibitor. Antiviral Res. *100*, 446–454. https://doi.org/10.1016/j.antiviral.2013.09.015.
16. Cross, R.W., Bornholdt, Z.A., Prasad, A.N., Geisbert, J.B., Borisevich, V., Agans, K.N., Deer, D.J., Melody, K., Fenton, K.A., Feldmann, H., et al. (2020). Prior vaccination with rVSV-ZEBOV does not interfere with but improves efficacy of postexposure antibody treatment. Nat. Commun. *11*, 3736. https://doi.org/10.1038/s41467-020-17446-4.
17. Qiu, X., Wong, G., Audet, J., Bello, A., Fernando, L., Alimonti, J.B., Fausther-Bovendo, H., Wei, H., Aviles, J., Hiatt, E., et al. (2014). Reversion of advanced Ebola virus disease in nonhuman primates with ZMapp. Nature *514*, 47–53. https://doi.org/10.1038/nature13777.
18. Gaudinski, M.R., Coates, E.E., Novik, L., Widge, A., Houser, K.V., Burch, E., Holman, L.A., Gordon, I.J., Chen, G.L., Carter, C., et al. (2019). Safety, tolerability, pharmacokinetics, and immunogenicity of the therapeutic monoclonal antibody mAb114 targeting Ebola virus glycoprotein (VRC 608): an open-label phase 1 study. Lancet *393*, 889–898. https://doi.org/10.1016/S0140-6736(19)30036-4.
19. Shepard, H.M., Phillips, G.L., D Thanos, C., and Feldmann, M. (2017). Developments in therapy with monoclonal antibodies and related proteins. Clin. Med. *17*, 220–232. https://doi.org/10.7861/clinmedicine.17-3-220.

20. Aboul-Ella, H., Gohar, A., Ali, A.A., Ismail, L.M., Mahmoud, A.E.E.R., Elkhatib, W.F., and Aboul-Ella, H. (2024). Monoclonal antibodies: From magic bullet to precision weapon. Mol. Biomed. *5*, 47. https://doi.org/10.1186/s43556-024-00210-1.

21. Nkuba-Ndaye, A., Dilu-Keti, A., Tovar-Sanchez, T., Diallo, M.S.K., Mukadi-Bamuleka, D., Kitenge, R., Formenty, P., Legand, A., Edidi-Atani, F., Thaurignac, G., et al. (2024). Effect of anti-Ebola virus monoclonal antibodies on endogenous antibody production in survivors of Ebola virus disease in the Democratic Republic of the Congo: an observational cohort study. Lancet Infect. Dis. *24*, 266–274. https://doi.org/10.1016/S1473-3099(23)00552-2.

22. Eisele, P., and Irvine, S. (2020). ERVEBO® (Ebola Zaire vaccine, live) Now Registered in Four African Countries, Within 90 Days of Reference Country Approval and WHO Prequalification. Democratic Republic of the Congo One of the First African Countries to Register ERVEBO. https://www.merck.com/news/ervebo-ebola-zaire-vaccine-live-now-registered-in-four-african-countries-within-90-days-of-reference-country-approval-and-who-prequalification/.

23. Anderson, E.M., and Coller, B.A.G. (2024). Translational success of fundamental virology: a VSV-vectored Ebola vaccine. J. Virol. *98*, e0162723. https://doi.org/10.1128/jvi.01627-23.

24. Huttner, A., Dayer, J.A., Yerly, S., Combescure, C., Auderset, F., Desmeules, J., Eickmann, M., Finckh, A., Goncalves, A.R., Hooper, J.W., et al. (2015). The effect of dose on the safety and immunogenicity of the VSV Ebola candidate vaccine: a randomised double-blind, placebo-controlled phase 1/2 trial. Lancet Infect. Dis. *15*, 1156–1166. https://doi.org/10.1016/S1473-3099(15)00154-1.

25. Ledgerwood, J.E., Sullivan, N.J., and Graham, B.S. (2015). Chimpanzee Adenovirus Vector Ebola Vaccine–Preliminary Report. N. Engl. J. Med. *373*, 776. https://doi.org/10.1056/NEJMc1505499.

26. Ewer, K., Rampling, T., Venkatraman, N., Bowyer, G., Wright, D., Lambe, T., Imoukhuede, E.B., Payne, R., Fehling, S.K., Strecker, T., et al. (2016). A Monovalent Chimpanzee Adenovirus Ebola Vaccine Boosted with MVA. N. Engl. J. Med. *374*, 1635–1646. https://doi.org/10.1056/NEJMoa1411627.

27. Muhlberger, E., Lotfering, B., Klenk, H.D., and Becker, S. (1998). Three of the four nucleocapsid proteins of Marburg virus, NP, VP35, and L, are sufficient to mediate replication and transcription of Marburg virus-specific monocistronic minigenomes. J. Virol. *72*, 8756–8764. https://doi.org/10.1128/JVI.72.11.8756-8764.1998.

28. Groseth, A., Feldmann, H., Theriault, S., Mehmetoglu, G., and Flick, R. (2005). RNA polymerase I-driven minigenome system for Ebola viruses. J. Virol. *79*, 4425–4433. https://doi.org/10.1128/JVI.79.7.4425-4433.2005.

29. Muhlberger, E., Weik, M., Volchkov, V.E., Klenk, H.-D., and Becker, S. (1999). Comparison of the Transcription and Replication Strategies of Marburg Virus and Ebola Virus by Using Artificial Replication Systems. J. Virol. *73*, 2333–2342. https://doi.org/10.1128/jvi.73.3.2333-2342.1999.

30. Hoenen, T., Groseth, A., de Kok-Mercado, F., Kuhn, J.H., and Wahl-Jensen, V. (2011). Minigenomes, transcription and replication competent virus-like particles and beyond: reverse genetics systems for filoviruses and other negative stranded hemorrhagic fever viruses. Antiviral Res. *91*, 195–208. https://doi.org/10.1016/j.antiviral.2011.06.003.

31. Feldmann, H., Sanchez, A., and Geisbert, T. (2013). Filoviridae: Marburg and ebola viruses. In Fields Virology (Wolters Kluwer Health Adis (ESP)).

32. Hoenen, T., Jung, S., Herwig, A., Groseth, A., and Becker, S. (2010). Both matrix proteins of Ebola virus contribute to the regulation of viral genome replication and transcription. Virology *403*, 56–66. https://doi.org/10.1016/j.virol.2010.04.002.

33. Hoenen, T., Groseth, A., Kolesnikova, L., Theriault, S., Ebihara, H., Hartlieb, B., Bamberg, S., Feldmann, H., Ströher, U., and Becker, S. (2006). Infection of naive target cells with virus-like particles: implications for the function of ebola virus VP24. J. Virol. *80*, 7260–7264. https://doi.org/10.1128/JVI.00051-06.

34. Bamberg, S., Kolesnikova, L., Möller, P., Klenk, H.D., and Becker, S. (2005). VP24 of Marburg virus influences formation of infectious particles. J. Virol. *79*, 13421–13433. https://doi.org/10.1128/JVI.79.21.13421-13433.2005.

35. Geisbert, T.W., Lee, A.C.H., Robbins, M., Geisbert, J.B., Honko, A.N., Sood, V., Johnson, J.C., de Jong, S., Tavakoli, I., Judge, A., et al. (2010). Postexposure protection of non-human primates against a lethal Ebola virus challenge with RNA interference: a proof-of-concept study. Lancet *375*, 1896–1905. https://doi.org/10.1016/S0140-6736(10)60357-1.

36. Geisbert, T.W., Hensley, L.E., Kagan, E., Yu, E.Z., Geisbert, J.B., Daddario-DiCaprio, K., Fritz, E.A., Jahrling, P.B., McClintock, K., Phelps, J.R., et al. (2006). Postexposure protection of guinea pigs against a lethal ebola virus challenge is conferred by RNA interference. J. Infect. Dis. *193*, 1650–1657. https://doi.org/10.1086/504267.

37. Miller, J.T., Khvorova, A., Scaringe, S.A., and Le Grice, S.F.J. (2004). Synthetic tRNALys,3 as the replication primer for the HIV-1HXB2 and HIV-1Mal genomes. Nucleic Acids Res. *32*, 4687–4695. https://doi.org/10.1093/nar/gkh813.

38. Scherer, L.J., Frank, R., and Rossi, J.J. (2007). Optimization and characterization of tRNA-shRNA expression constructs. Nucleic Acids Res. *35*, 2620–2628. https://doi.org/10.1093/nar/gkm103.

39. Avcilar-Kucukgoze, I., and Kashina, A. (2020). Hijacking tRNAs From Translation: Regulatory Functions of tRNAs in Mammalian Cell Physiology. Front. Mol. Biosci. *7*, 610617. https://doi.org/10.3389/fmolb.2020.610617.

40. Yuan, Y., Hwang, E.S., and Altman, S. (1992). Targeted cleavage of mRNA by human RNase P. Proc. Natl. Acad. Sci. USA *89*, 8006–8010. https://doi.org/10.1073/pnas.89.17.8006.

41. Rossmanith, W. (2012). Of P and Z: mitochondrial tRNA processing enzymes. Biochim. Biophys. Acta *1819*, 1017–1026. https://doi.org/10.1016/j.bbagrm.2011.11.003.

42. Carthew, R.W., and Sontheimer, E.J. (2009). Origins and Mechanisms of miRNAs and siRNAs. Cell *136*, 642–655. https://doi.org/10.1016/j.cell.2009.01.035.

43. Watt, A., Moukambi, F., Banadyga, L., Groseth, A., Callison, J., Herwig, A., Ebihara, H., Feldmann, H., and Hoenen, T. (2014). A Novel Life Cycle Modeling System for Ebola Virus Shows a Genome Length-Dependent Role of VP24 in Virus Infectivity. J. Virol. *88*, 10511–10524. https://doi.org/10.1128/Jvi.01272-14.

44. Zell, B.N.S.,V.A., Lu, S.-C., Wang, L., Barry, M.A., Ebihara, H., and Yamaoka, S. (2024). Development of a Pentacistronic Ebola Virus Minigenome and Its Application to Model Mouse-Adapted Ebola Virus. Preprints *2024*, 2024120940. https://doi.org/10.20944/preprints202412.0940.v1.

45. Batra, J., Hultquist, J.F., Liu, D., Shtanko, O., Von Dollen, J., Satkamp, L., Jang, G.M., Luthra, P., Schwarz, T.M., Small, G.I., et al. (2018). Protein Interaction Mapping Identifies RBBP6 as a Negative Regulator of Ebola Virus Replication. Cell *175*, 1917–1930.e13. https://doi.org/10.1016/j.cell.2018.08.044.

46. Batra, J., Mori, H., Small, G.I., Anantpadma, M., Shtanko, O., Mishra, N., Zhang, M., Liu, D., Williams, C.G., Biedenkopf, N., et al. (2021). Non-canonical proline-tyrosine interactions with multiple host proteins regulate Ebola virus infection. EMBO J. *40*, e105658. https://doi.org/10.15252/embj.2020105658.

47. Welch, S.R., Spengler, J.R., Harmon, J.R., Coleman-McCray, J.D., Scholte, F.E.M., Genzer, S.C., Lo, M.K., Montgomery, J.M., Nichol, S.T., and Spiropoulou, C.F. (2022). Defective Interfering Viral Particle Treatment Reduces Clinical Signs and Protects Hamsters from Lethal Nipah Virus Disease. mBio *13*, e0329421. https://doi.org/10.1128/mbio.03294-21.

48. Welch, S.R., Tilston, N.L., Lo, M.K., Whitmer, S.L.M., Harmon, J.R., Scholte, F.E.M., Spengler, J.R., Duprex, W.P., Nichol, S.T., and Spiropoulou, C.F. (2020). Inhibition of Nipah Virus by Defective Interfering Particles. J. Infect. Dis. *221*, S460–S470. https://doi.org/10.1093/infdis/jiz564.

49. Bhat, T., Cao, A., and Yin, J. (2022). Virus-like Particles: Measures and Biological Functions. Viruses *14*, 383. https://doi.org/10.3390/v14020383.

50. Chaturvedi, S., Vasen, G., Pablo, M., Chen, X., Beutler, N., Kumar, A., Tanner, E., Illouz, S., Rahgoshay, D., Burnett, J., et al. (2021). Identification of a therapeutic interfering particle-A single-dose SARS-CoV-2 antiviral intervention with a high barrier to resistance. Cell *184*, 6022–6036.e18. https://doi.org/10.1016/j.cell.2021.11.004.

51. Rezelj, V.V., Carrau, L., Merwaiss, F., Levi, L.I., Erazo, D., Tran, Q.D., Henrion-Lacritick, A., Gausson, V., Suzuki, Y., Shengjuler, D., et al. (2021). Defective viral genomes as therapeutic interfering particles against flavivirus infection in mammalian and mosquito hosts. Nat. Commun. *12*, 2290.

52. Tanner, E.J., Jung, S.-Y., Glazier, J., Thompson, C., Zhou, Y., Martin, B., Son, H.-I., Riley, J.L., and Weinberger, L.S. (2019). Discovery and Engineering of a Therapeutic Interfering Particle (TIP): a combination self-renewing antiviral. Preprint at bioRxiv. https://doi.org/10.1101/820456.

53. Bwire, G., Sartorius, B., Guerin, P., Tegegne, M.A., Okware, S.I., and Talisuna, A.O. (2023). Sudan Ebola virus (SUDV) outbreak in Uganda, 2022: lessons learnt and future priorities for sub-Saharan Africa. BMC Med. *21*, 144. https://doi.org/10.1186/s12916-023-02847-1.

54. Okware, S.I., Omaswa, F.G., Zaramba, S., Opio, A., Lutwama, J.J., Kamugisha, J., Rwaguma, E.B., Kagwa, P., and Lamunu, M. (2002). An outbreak of Ebola in Uganda. Trop. Med. Int. Health *7*, 1068–1075. https://doi.org/10.1046/j.1365-3156.2002.00944.x.

55. World Health Organization (2023). Disease Outbreak News; Marburg virus disease - the United Republic of Tanzania. https://www.who.int/emergencies/disease-outbreak-news/item/2023-DON471.

56. World Health Organization (2021). Disease Outbreak News; Marburg virus disease-Guinea. https://www.who.int/emergencies/disease-outbreak-news/item/2021-DON331.

57. Boehmann, Y., Enterlein, S., Randolf, A., and Mühlberger, E. (2005). A reconstituted replication and transcription system for Ebola virus Reston and comparison with Ebola virus Zaire. Virology *332*, 406–417. https://doi.org/10.1016/j.virol.2004.11.018.

58. Kamper, L., Zierke, L., Schmidt, M.L., Muller, A., Wendt, L., Brandt, J., Hartmann, E., Braun, S., Holzerland, J., Groseth, A., and Hoenen, T. (2019). Assessment of the function and intergenus-compatibility of Ebola and Lloviu virus proteins. J. Gen. Virol. *100*, 760–772. https://doi.org/10.1099/jgv.0.001261.

59. Manhart, W.A., Pacheco, J.R., Hume, A.J., Cressey, T.N., Deflubé, L.R., and Mühlberger, E. (2018). A Chimeric Lloviu Virus Minigenome System Reveals that the Bat-Derived Filovirus Replicates More Similarly to Ebolaviruses than Marburgviruses. Cell Rep. *24*, 2573–2580.e4. https://doi.org/10.1016/j.celrep.2018.08.008.

60. He, F., Melen, K., Maljanen, S., Lundberg, R., Jiang, M., Osterlund, P., Kakkola, L., and Julkunen, I. (2017). Ebolavirus protein VP24 interferes with innate immune responses by inhibiting interferon-lambda1 gene expression. Virology *509*, 23–34. https://doi.org/10.1016/j.virol.2017.06.002.

61. Mateo, M., Reid, S.P., Leung, L.W., Basler, C.F., and Volchkov, V.E. (2010). Ebolavirus VP24 binding to karyopherins is required for inhibition of interferon signaling. J. Virol. *84*, 1169–1175. https://doi.org/10.1128/JVI.01372-09.

62. Reid, S.P., Leung, L.W., Hartman, A.L., Martinez, O., Shaw, M.L., Carbonnelle, C., Volchkov, V.E., Nichol, S.T., and Basler, C.F. (2006). Ebola virus VP24 binds karyopherin alpha1 and blocks STAT1 nuclear accumulation. J. Virol. *80*, 5156–5167. https://doi.org/10.1128/JVI.02349-05.

63. Schwarz, T.M., Edwards, M.R., Diederichs, A., Alinger, J.B., Leung, D.W., Amarasinghe, G.K., and Basler, C.F. (2017). VP24-Karyopherin Alpha Binding Affinities Differ between Ebolavirus Species, Influencing Interferon Inhibition and VP24 Stability. J. Virol. *91*, e01715-16. https://doi.org/10.1128/JVI.01715-16.

64. Cross, R.W., Mire, C.E., Feldmann, H., and Geisbert, T.W. (2018). Post-exposure treatments for Ebola and Marburg virus infections. Nat. Rev. Drug Discov. *17*, 413–434. https://doi.org/10.1038/nrd.2018.73.

65. Basler, C.F., Krogan, N.J., Leung, D.W., and Amarasinghe, G.K. (2019). Virus and host interactions critical for filoviral RNA synthesis as therapeutic targets. Antivir. Res. *162*, 90–100. https://doi.org/10.1016/j.antiviral.2018.12.006.

66. Akinc, A., Maier, M.A., Manoharan, M., Fitzgerald, K., Jayaraman, M., Barros, S., Ansell, S., Du, X., Hope, M.J., Madden, T.D., et al. (2019). The Onpattro story and the clinical translation of nanomedicines containing nucleic acid-based drugs. Nat. Nanotechnol. *14*, 1084–1087. https://doi.org/10.1038/s41565-019-0591-y.

67. Akinc, A., Querbes, W., De, S., Qin, J., Frank-Kamenetsky, M., Jayaprakash, K.N., Jayaraman, M., Rajeev, K.G., Cantley, W.L., Dorkin, J.R., et al. (2010). Targeted delivery of RNAi therapeutics with endogenous and exogenous ligand-based mechanisms. Mol. Ther. *18*, 1357–1364. https://doi.org/10.1038/mt.2010.85.

68. Dunning, J., Sahr, F., Rojek, A., Gannon, F., Carson, G., Idriss, B., Massaquoi, T., Gandi, R., Joseph, S., Osman, H.K., et al. (2016). Experimental Treatment of Ebola Virus Disease with TKM-130803: A Single-Arm Phase 2 Clinical Trial. PLoS Med. *13*, e1001997. https://doi.org/10.1371/journal.pmed.1001997.

69. Everton, E., Rizvi, F., Smith, A.R., Beattie, M., Tam, Y., Pardi, N., Weissman, D., and Gouon-Evans, V. (2021). Transient yet Robust Expression of Proteins in the Mouse Liver via Intravenous Injection of Lipid Nanoparticle-encapsulated Nucleoside-modified mRNA. Bio. Protoc. *11*, e4184. https://doi.org/10.21769/BioProtoc.4184.

70. Thi, E.P., Mire, C.E., Ursic-Bedoya, R., Geisbert, J.B., Lee, A.C.H., Agans, K.N., Robbins, M., Deer, D.J., Fenton, K.A., MacLachlan, I., and Geisbert, T.W. (2014). Marburg virus infection in nonhuman primates: Therapeutic treatment by lipid-encapsulated siRNA. Sci. Transl. Med. *6*, 250ra116. https://doi.org/10.1126/scitranslmed.3009706.

71. Thi, E.P., Mire, C.E., Lee, A.C.H., Geisbert, J.B., Zhou, J.Z., Agans, K.N., Snead, N. M., Deer, D.J., Barnard, T.R., Fenton, K.A., et al. (2015). Lipid nanoparticle siRNA treatment of Ebola-virus-Makona-infected nonhuman primates. Nature *521*, 362–365. https://doi.org/10.1038/nature14442.

72. Scott, J.T., Sharma, R., Meredith, L.W., Dunning, J., Moore, C.E., Sahr, F., Ward, S., Goodfellow, I., and Horby, P.; RAPIDE-TKM trial team (2020). Pharmacokinetics of TKM-130803 in Sierra Leonean patients with Ebola virus disease: plasma concentrations exceed target levels, with drug accumulation in the most severe patients. EBioMedicine *52*, 102601. https://doi.org/10.1016/j.ebiom.2019.102601.

73. Brian, J., Parrett, S.Y., and Barry, M.A. (2024). Reducing Off-target Expression of mRNA Therapeutics and Vaccines in the Liver with MicroRNA Binding Sites. Mol. Ther. Methods Clin. Dev. *33*, 101402. https://doi.org/10.1016/j.omtm.2024.101402.

74. Chander, N., Basha, G., Yan Cheng, M.H., Witzigmann, D., and Cullis, P.R. (2023). Lipid nanoparticle mRNA systems containing high levels of sphingomyelin engender higher protein expression in hepatic and extra-hepatic tissues. Mol. Ther. Methods Clin. Dev. *30*, 235–245. https://doi.org/10.1016/j.omtm.2023.06.005.

75. Kim, M., Jeong, M., Hur, S., Cho, Y., Park, J., Jung, H., Seo, Y., Woo, H.A., Nam, K. T., Lee, K., and Lee, H. (2021). Engineered ionizable lipid nanoparticles for targeted delivery of RNA therapeutics into different types of cells in the liver. Sci. Adv. *7*, eabf4398. https://doi.org/10.1126/sciadv.abf4398.

76. Johnson, E., Jaax, N., White, J., and Jahrling, P. (1995). Lethal experimental infections of rhesus monkeys by aerosolized Ebola virus. Int. J. Exp. Pathol. *76*, 227–236.

77. Murphy, F.A., Simpson, D.I., Whitfield, S.G., Zlotnik, I., and Carter, G.B. (1971). Marburg virus infection in monkeys. Ultrastructural studies. Lab. Invest. *24*, 279–291.

78. Davis, K.J., Anderson, A.O., Geisbert, T.W., Steele, K.E., Geisbert, J.B., Vogel, P., Connolly, B.M., Huggins, J.W., Jahrling, P.B., and Jaax, N.K. (1997). Pathology of experimental Ebola virus infection in African green monkeys. Involvement of fibroblastic reticular cells. Arch. Pathol. Lab Med. *121*, 805–819.

79. Ryabchikova, E., Strelets, L., Kolesnikova, L., Pyankov, O., and Sergeev, A. (1996). Respiratory Marburg virus infection in guinea pigs. Arch. Virol. *141*, 2177–2190. https://doi.org/10.1007/BF01718224.

80. Ryabchikova, E., Kolesnikova, L., Smolina, M., Tkachev, V., Pereboeva, L., Baranova, S., Grazhdantseva, A., and Rassadkin, Y. (1996). Ebola virus infection in guinea pigs: presumable role of granulomatous inflammation in pathogenesis. Arch. Virol. *141*, 909–921. https://doi.org/10.1007/BF01718165.

81. Ryabchikova, E.I., Kolesnikova, L.V., and Luchko, S.V. (1999). An analysis of features of pathogenesis in two animal models of Ebola virus infection. J. Infect. Dis. *179*, S199–S202. https://doi.org/10.1086/514293.

82. Jaax, N.K., Davis, K.J., Geisbert, T.J., Vogel, P., Jaax, G.P., Topper, M., and Jahrling, P.B. (1996). Lethal experimental infection of rhesus monkeys with Ebola-Zaire (Mayinga) virus by the oral and conjunctival route of exposure. Arch. Pathol. Lab Med. *120*, 140–155.

83. Welch, S.R., Spengler, J.R., Genzer, S.C., Coleman-McCray, J.D., Harmon, J.R., Sorvillo, T.E., Scholte, F.E.M., Rodriguez, S.E., O'Neal, T.J., Ritter, J.M., et al. (2023). Single-dose mucosal replicon-particle vaccine protects against lethal Nipah virus infection up to 3 days after vaccination. Sci. Adv. *9*, eadh4057. https://doi.org/10.1126/sciadv.adh4057.

84. Singh, K., Marasini, B., Chen, X., Ding, L., Wang, J.J., Xiao, P., Villinger, F., and Spearman, P. (2020). A Bivalent, Spherical Virus-Like Particle Vaccine Enhances Breadth of Immune Responses against Pathogenic Ebola Viruses in Rhesus Macaques. J. Virol. *94*, e01884-19. https://doi.org/10.1128/JVI.01884-19.

85. Warfield, K.L., Bosio, C.M., Welcher, B.C., Deal, E.M., Mohamadzadeh, M., Schmaljohn, A., Aman, M.J., and Bavari, S. (2003). Ebola virus-like particles protect from lethal Ebola virus infection. Proc. Natl. Acad. Sci. USA *100*, 15889–15894. https://doi.org/10.1073/pnas.2237038100.

86. Malherbe, D.C., Domi, A., Hauser, M.J., Atyeo, C., Fischinger, S., Hyde, M.A., Williams, J.M., Alter, G., Guirakhoo, F., and Bukreyev, A. (2022). A single immunization with a modified vaccinia Ankara vectored vaccine producing Sudan virus-like particles protects from lethal infection. NPJ Vaccines *7*, 83. https://doi.org/10.1038/s41541-022-00512-x.

87. Warfield, K.L., Swenson, D.L., Olinger, G.G., Kalina, W.V., Aman, M.J., and Bavari, S. (2007). Ebola Virus-Like Particle-Based Vaccine Protects Nonhuman Primates against Lethal Ebola Virus Challenge. J. Infect. Dis. *196*, S430–S437. https://doi.org/10.1086/520583.

88. Shen, W., Liu, S., and Ou, L. (2022). rAAV immunogenicity, toxicity, and durability in 255 clinical trials: A meta-analysis. Front. Immunol. *13*, 1001263. https://doi.org/10.3389/fimmu.2022.1001263.

OMTN, Volume *36*

Supplemental information

Infection-sensing minigenome as a novel therapeutic approach against Ebola virus

Lin Wang, Brady N. Zell, Brian J. Parrett, Michael A. Barry, and Satoko Yamaoka

A.

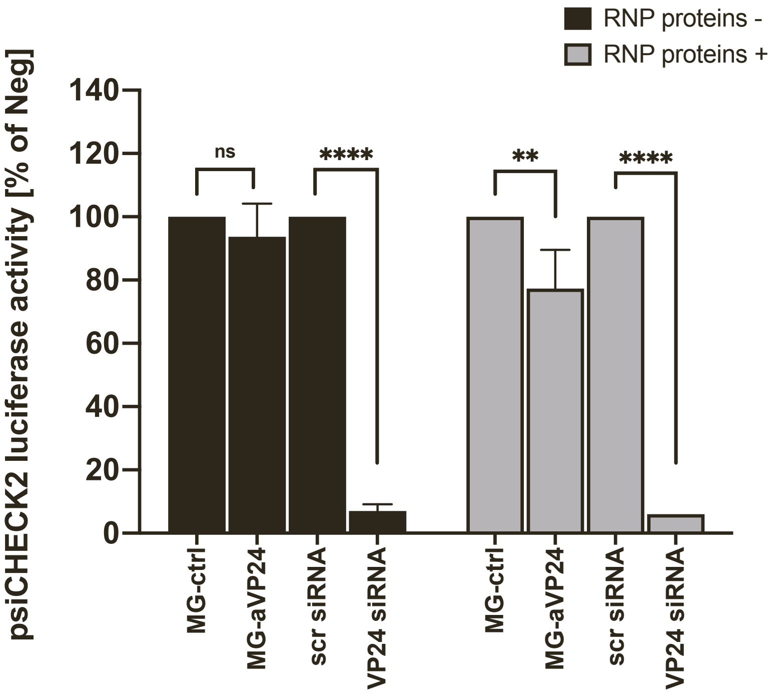

RNP proteins -
RNP proteins +
psiCHECK2 luciferase activity [% of Neg]
140
120
100
80
60
40
20
0
ns
****
**
****
MG-ctrl
MG-aVP24
scr siRNA
VP24 siRNA
MG-ctrl
MG-aVP24
scr siRNA
VP24 siRNA

B.

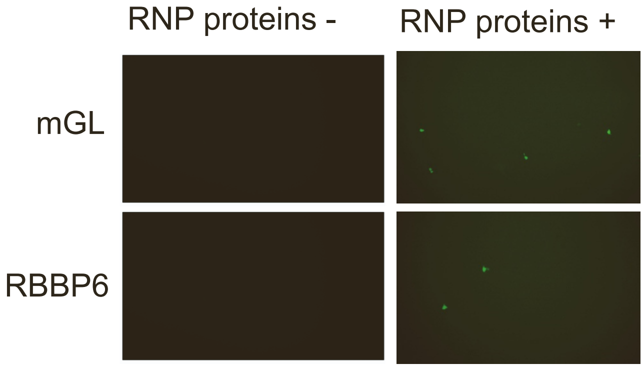

RNP proteins -
RNP proteins +
mGL
RBBP6

Fig. S1. Stability of therapeutic RNAs. A) The IVT 1cis-MG-ctrl and 1cis-MG-aVP24 RNAs, scramble and anti-VP24 siRNAs, were transfected into 293 cells 24 hours before the transfection of EBOV helper plasmids (pCAGGS-L, VP30, VP35 and NP). Six hours after helper plasmids transfection, the psiCHECK2-VP24 plasmid was introduced to evaluate the silencing of VP24. Luciferase activity was measured 24 hours post-transfection. *Renilla* luciferase activity was normalized to firefly luciferase activity. Data are shown as mean percentage reduction of luciferase activity (*Renilla*/firefly) relative to each negative control, set at 100%. Error bars indicate the SD of three independent biological replicates. Ordinary One-way ANOVA was used for statistical analysis: ns: $P > 0.05$, ** $P \leq 0.01$, **** $P \leq 0.0001$. **B**) IVT 1cis-MG-mGL and 1cis-MG-mGL-RBBP6 RNAs were transfected into 293 cells 24 hours prior to EBOV helper plasmids transfection. Fluorescence images (original magnification x250) were taken 24 hours after helper plasmids transfection.
